# Neo-Domestication of an Interspecific Tetraploid *Helianthus annuus* × *Helianthus tuberous* Population That Segregates for Perennial Habit

**DOI:** 10.3390/genes9090422

**Published:** 2018-08-21

**Authors:** Michael B. Kantar, Sariel Hüber, Adam Herman, Dan G. Bock, Greg Baute, Kevin Betts, Matthew Ott, Yaniv Brandvain, Donald Wyse, Robert M. Stupar, Loren H. Rieseberg

**Affiliations:** 1 Department of Tropical Plant & Soil Sciences, St. John Plant Science Lab, Room 102, 3190 Maile Way, Honolulu, HI 96822, USA; 2 Biodiversity Research Centre and Department of Botany, University of British Columbia, 3529-6270 University Boulevard, Vancouver, British Columbia, BC V6T 1Z4, Canada; sarielh@migal.org.il (S.H.); dan.g.bock@gmail.com (D.G.B.); gregbaute@gmail.com (G.B.); lriesebe@mail.ubc.ca (L.H.R.); 3 Department of Agronomy and Plant Genetics, University of Minnesota, 411 Borlaug Hall, 1991 Upper Buford Circle, St. Paul, MN 55108, USA; betts003@umn.edu (K.B.); ottxx142@umn.edu (M.O.); wysex001@umn.edu (D.W.); stup0004@umn.edu (R.M.S.); 4 Department of Biotechnology, Tel-Hai Academic College, Upper Galilee 12210, Israel; 5 MIGAL—Galilee Research Institute, Kiryat Shmona 11016, Israel; 6 Department of Plant and Microbial Biology, 123 Snyder Hall, 1475 Gortner Ave, Saint Paul, MN 55108, USA; aherman@umn.edu (A.H.); ybrandva@umn.edu (Y.B.)

**Keywords:** domestication syndrome, sustainable agriculture, rapid evolution, perenniality

## Abstract

Perennial agriculture has been proposed as an option to improve the sustainability of cropping systems, by increasing the efficiency of resource use, while also providing ecosystem services. Neo-domestication, the contemporary domestication of plants that have not previously been used in agriculture, can be used to generate new crops for these systems. Here we explore the potential of a tetraploid (2n = 4x = 68) interspecific hybrid sunflower as a perennial oilseed for use in multifunctional agricultural systems. A population of this novel tetraploid was obtained from crosses between the annual diploid oilseed crop *Helianthus annuus* (2n = 2x = 34) and the perennial hexaploid tuber crop *Helianthus tuberosus* (2n = 6x = 102). We selected for classic domestication syndrome traits for three generations. Substantial phenotypic gains were made, in some cases approaching 320%. We also analyzed the genetic basis of tuber production (i.e., perenniality), with the goal of obtaining molecular markers that could be used to facilitate future breeding in this system. Results from quantitative trait locus (QTL) mapping suggest that tuber production has an oligogenic genetic basis. Overall, this study indicates that substantial gains towards domestication goals can be achieved over contemporary time scales.

## 1. Introduction

Over the past decade, increased interest in sustainable agricultural systems has motivated an intensification of research on perennial crops [1,2,3]. Combined with recent advances in DNA sequencing, this has led to an expanding number of plant species being considered as candidate perennial crops [4,5,6,7]. Under this framework, an issue of primary importance is the length of time required for the domestication process to occur. Historically, this has been a controversial topic with multiple methods used to explore the question providing contradictory results [8,9,10,11,12,13,14]. In some cases, domestication traits are thought to have reached fixation very quickly (i.e., in decades [15], although the process is generally thought to have occurred over protracted time scales (i.e., over millennia; [16]).

The renewal of the debate surrounding the length of the domestication process has sparked a re-evaluation of the concept of domestication ideotypes (i.e., the ideal domesticated phenotypes, that are actively pursued through breeding [17]. Specifically, there is great interest in knowing whether the domestication of new plant material for new ideotypes could be accomplished in a short timeframe [18]. Classic understanding of domestication comes from annual grasses (*Poaceae*; [19,20,21,22,23]), which contribute more than 1/3 of the world’s calories [24,25]. Grasses were used to define the ‘domestication syndrome’, which includes traits such as non-shattering and modification of plant architecture (e.g., reduced height and branching) and phenology (e.g., loss of seed dormancy and synchronous flowering [19]). This grass-centered ‘domestication syndrome’ has been used to define the ideotype of modern agriculture, that focuses on highly productive (high-yielding) varieties, and labor-saving cropping systems, which has been accentuated as modern agriculture has shifted towards mechanization [26]. However, new definitions of productivity that include sustainability [1,27,28,29] have led to exploration of alternative crop ideotypes that combine yield with ecosystem services. For example, perennial plants, such as intermediate wheatgrass (*Thinopyrum intermedium*), have multiple uses (e.g., food and ecosystem services) that may not be a part of conventional domestication ideotypes (e.g., extensive root systems, extra floral nectaries, secondary metabolites), but that may increase the crop value by creating ecosystem services in addition to traditional value. These different plant forms have led to the idea that the ideotype for most productive domesticates may not have been recovered [1,17,30].

In sunflower, efforts have centered around developing one such perennial ideotype. Its characteristics include high seed yield (high seed weight and large head size), favorable plant architecture traits (i.e., reduced branching and one central head), as well as a perennial habit [31] that can be used for more sustainable production [32]. To meet this goal, an interspecific hybrid between the annual oilseed crop *Helianthus annuus* (2n = 2x = 34) and the perennial tuber crop *Helianthus tuberosus* was developed (2n = 6x = 102 [31,32]. When *H. annuus* × *H. tuberosus* hybrids were allowed to intermate, they segregated for perennial organ (rhizomes and tubers) formation, with tuber production being a better predictor of perenniality [31]. Here we explore the development of this new perennial crop through selection on domestication syndrome traits. Furthermore, we use genotyping-by-sequencing (GBS [33,34]) to generate molecular markers to study the genetic basis of perenniality and to facilitate downstream marker-assisted selection.

## 2. Materials and Methods

### 2.1. Populations Used and Selection within the Perennial Sunflower Breeding Program

The perennial sunflower breeding program was initiated between 2003–2006, from an initial population of interspecific F_1_ hybrids between *H. annuus* and *H. tuberosus* [32]. Crosses were made using 18 *H. tuberosus* (perennial) parents which were wild collected from Rosemount Minnesota (MN, USA) and three inbred *H. annuus* (annual) lines: HA89 (released by the USDA-ARS in 1971), CMS HA89-PET1 (cytoplasmic male sterile version of the accession HA89 using *Helianthus petiolaris* cytoplasm), and HA434 [35]. The HA 89 (male fertile version) and HA 434 lines were used as male parents and CMS HA89-PET1 was used as a female parent. Parents and F_1_ populations were previously phenotyped for perenniality and domestication syndrome traits including seed size, branching, pollen fertility, head diameter, and number of heads [31]. Specifically, branching type was scored on a scale of 0–4 according to Hockett and Knowles [36], with 0 being no branching, head number was determined by counting the number of heads for each plant at physiological maturity, maximum head diameter (cm) was measured in cm after plant physiological maturity, average head diameter (cm) was measured in cm after plant physiological maturity with ten randomly selected heads (unless the plant had fewer), including the central head, were measured to calculate average head diameter, number of seeds per head was calculated by dividing the total number of seeds by the number of heads harvested, seed weight (grams) was calculated by threshing ten (or maximum number) of random heads from each plant, including the central head, and weighing the resulting seeds, and individual seed weight (grams) was calculated by weighing the seed from the ten heads and dividing by the total number of seed.

Based on initial observations from Kantar et al. [31], we developed a selection index for domestication syndrome traits. The selection index was developed to attempt to move the population toward the perennial sunflower ideotype. This index was calculated as follows:

index=largest head diameter+average head diameter−number of heads+(2×total seed number)+individual seed weight

We used this index to select on standardized phenotypes for three generations on maternal half-sib families that made up each breeding line, F_1_, Intermated F_1_ generation 1 (IM_1_F_1_), and Intermated F_1_ generation 2 (IM_2_F_1_). The top twenty percent of the maternal half sib families were selected for inclusion in the next generation of plant material. Gain was calculated based on the phenotypic values of each maternal half-sib family and by comparing both the average of each generation and best half-sib family in each generation.

Breeding populations for this study, which consisted of the IM_1_F_1_ population created in 2010 and the IM_2_F_1_ population created in 2011, were phenotyped across four years. Specifically, phenotyping was performed in Rosemount (MN, USA) in 2011 and 2012 in un-replicated field plots, and in St. Paul (MN, USA) in 2013 and 2014, in a randomized complete block with three replications in 4 ft × 4 ft plots. Selections used in this study can be found in Table S1. Significance between generations was identified by created 95% confidence interval around all plant from each generation based on the replicated trials. We combined data from all breeding trials to explore gain from selection for domestication syndrome traits. We built linear models using the R package lme4 version 1.1.15 to calculate heritability and best linear unbiased prediction (BLUP) to assess improvement in domestication syndrome traits [37].

### 2.2. Tuber Phenotyping

Tuber production is an important perenniality trait that segregates in the intermated populations. Identifying marker trait associations would allow for marker-assisted selection to be used, thereby facilitating the breeding program. For example, with such markers, tuber-producing plants could be identified before physiological maturity (e.g., before physiological stage R-9 for sunflower, usually recognized based on the production of brittle and brown phyllaries; [38]), when tubers are commonly harvested. To explore the association between markers and tuber production, we used an intermated F_1_ (IM_1_F_1_) population. The population was randomly intermated via open pollination of an initial F_1_ population developed in 2007 by Hulke and Wyse [32]). During the winter of 2010–2011, 151 IM_1_F_1_ plants were grown in the greenhouse in St. Paul and screened for tuber production (presence/absence), along with control wild-collected *H. tuberosus* plants. *H. tuberosus* controls were used to ensure greenhouse conditions were sufficient to induce tuberization. For all plants, we used 30 cm pots with 50–50 mix of Sunshine professional growing mix^®^ (Sun Gro (headquartered in Agawam, MA, USA) and soil. Growth conditions consisted of 14 h day-length at 24 °C. Plants were grown to physiological maturity (equivalent of stage R-9, as described above).

### 2.3. DNA Extraction and Genotyping-by-Sequencing Library Preparation

Of the population that segregated for tuber production (151 IM_1_F_1_ plants), genomic DNA was isolated from fresh or freeze-dried leaf tissue for 96 individuals (14 parents (including one technical duplicate of HA89) and 81 IM_1_F_1_) using either a Qiagen Plant DNeasy Mini kit according to the manufacturer’s protocol (Qiagen, Valencia, CA, USA), or using a modified CTAB (Cetyl trimethylammonium bromide) procedure optimized for sunflower [39]. DNA yield was assessed on a Qubit 2.0 Fluorometer (Thermo Fisher Scientific, Waltham, MA, USA). Genotyping-by-sequencing libraries were prepared according to Elshire et al. [33]. Briefly, DNA was digested using PstI (New England Biolabs Inc., Ipswich, MA, USA). Adaptor barcodes were ligated onto digested DNA. The ligated fragments were then pooled, and PCR-amplified. GBS libraries were pair-end sequenced (2 × 100 bp reads) on one lane of an Illumina HiSeq 2000 (manufactured in San Diego, CA, USA), at the University of British Columbia Biodiversity Research Centre. All raw fastq files were deposited in the NCBI sequence read archive (SRA) under the accession number SRP127977.

### 2.4. Variant Identification and Marker Discovery

Sequences were demultiplexed into individual fastq files using a custom perl script that also trims the barcode and adapter sequences. Demultiplexed reads were aligned to the two draft reference genomes currently available for *H. annuus* (HA412-HO and XRQ; [40]) using BWA (Burrows-Wheeler Aligner) at default parameters (version 0.7.1 [41]). Finally, we called SNPs using the GATK Unified Genotyper using a tetrapoloid setting [42]. From the F_1_ mapping population, we excluded eight samples that were contaminated with DNA from another species due to duplicate barcode usage.

### 2.5. Quantitative Trait Loci Mapping

Variant call format (VCF) files were filtered for coverage (10 reads) and minor allele frequency (10%) using vcftools [43]. Also, we removed indels, keeping only bi-allelic SNPs. Markers were then filtered for missing data (<30%) and segregation distortion at *p* < 0.05 using a Chi-square test. Expected segregation differed for each marker, as the mapping population structure was complex, with multiple small half-sib families in a polyploid background. This meant that markers that did not conform to an expected ratio (e.g., 1:1, 1:2:1, 3:1) could not be reliably used, and were removed. The development of a linkage map was challenging for our population because of this unusual structure, and few linkage groups were resolved. To address this, we employed the marker positions on the *H. annuus* reference genome assembly as an initial hypothesis of marker locations, refining position based on the present population, creating a framework for the quantitative trait locus (QTL) analysis. This approach allows exploration of the subgenome of *H. tuberosus* that pairs with *H. annuus* in the interspecific hybrid. This subgenome is likely where segregation for perennial habit occurs. To remove markers that were incorrectly mapped (e.g., because of paralogy), we employed an linkage disequilibrium (LD) filter (as measured by D’ calculated in the R package genetics [44]), in which adjacent markers with D’ < 0.1, or markers with D’ < 0.05 against all other markers on a chromosome, were discarded. Lastly, we employed R/qtl’s [45], first we reformed linkage groups with recombination fraction of 0.3 and minimum lod of seven, then we used ‘Ripple’ function to validate/correct marker orders using a window of four markers and an error probability of 0.01. QTL mapping of tuber presence/absence was conducted using the Haley-Knott regression method as implemented in R/qtl, using the ‘binary’ phenotype option [46]. Statistical significance of QTLs was established by permutation tests [47], also implemented in R/qtl.

## 3. Results

### 3.1. Selection for Domestication Syndrome Traits

After initial evaluations of multiple breeding methods [31], a domestication approach among interspecific hybrids was adopted. We conducted selection for domestication syndrome traits in interspecific hybrid populations. Each generation included a round of intermating of the best half-sib families where only the maternal parent was controlled. Selection focused on classic domestication syndrome traits, such as changes in flowering time, increased head size, and increased seed size. These domestication syndrome traits increase utility of the plants, and move the material closer to the cultivated ideotype of annual sunflower.

**Figure 1:**
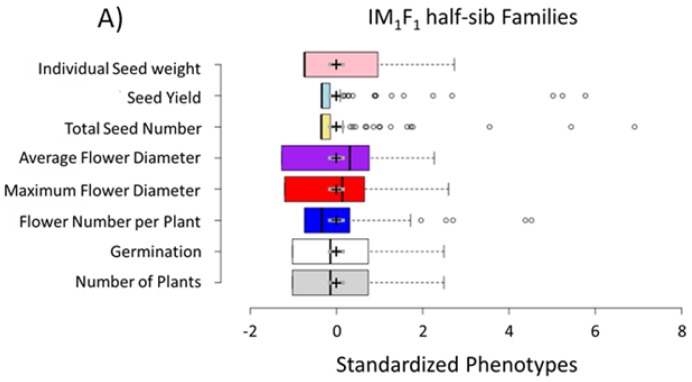

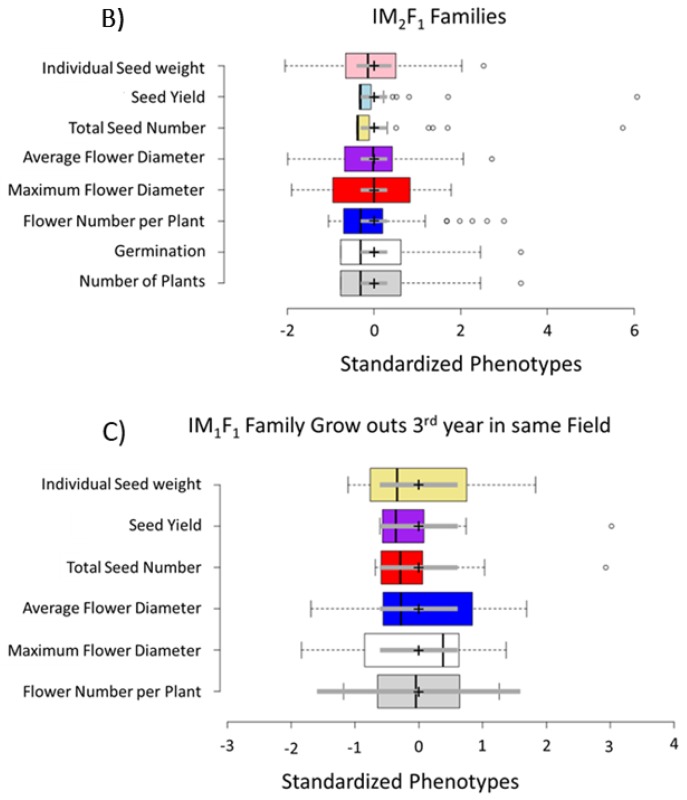
Phenotypic ranges are shown as standardized phenotypes in (**A**) IM_1_F_1_ half-sib families in the first two years; (**B**) IM_2_F_1_ families in the first year; and (**C**) IM_1_F_1_ families, evaluated as the third year after being in the same plots for three consecutive years. In the boxplots the center lines show the medians and box limits indicate the 25th and 75th percentiles. The whiskers extend 1.5 times the interquartile range from the 25th and 75th percentiles. The outliers are represented by dots and the crosses represent sample means

There was substantial phenotypic variation within every generation tested and year examined, which is expected due to the large number of loci segregating within the multi-parent interspecific domestication population. The phenotypic variation was extreme, reaching up to six standard deviations around the mean (Figure 1). There was substantial variation in plants grown clonally over multiple years in the same plots. However, yield generally decreased and plants exhibited less of a domestication phenotype when regrown in the same plot from tubers rather than when reseeded. Plants in multiyear plots also exhibited fewer extreme domestication syndrome phenotypes (Figure 1c). In a principal component analysis of selected phenotypes, seeds per head contributed most to the first principal component (43.99% variance explained).

**Figure 2:**
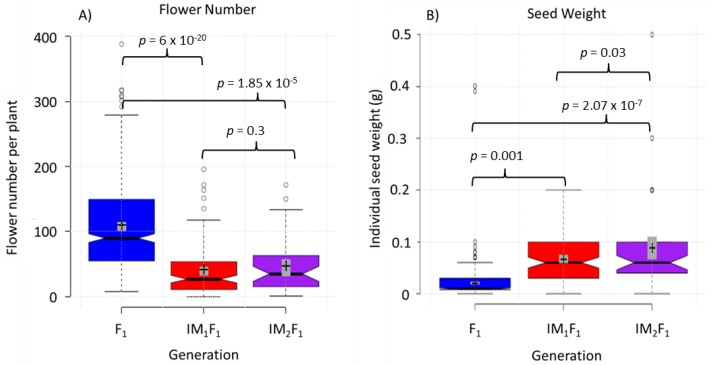

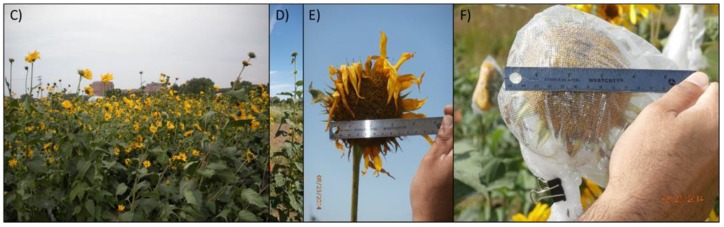
Gain from selection in domestication traits in the interspecific hybrid generation and subsequent generations derived from intermating within the population. (**A**) Decrease in flower number; (**B**) Increase in individual seed weight; (**C**) Multi-branched small headed F_1_ populations, (**D**) Minimally branched IM_1_F_1_, (**E**) IM_1_F_1_ with increased head size, (**F**) IM_1_F_1_ with increased head size. *P*-values represent *t*-tests between different generations.

The first steps of domestication were initiated through selection based on an index that involved multiple important domestication characters. While this multi-trait approach may limit selection efficiency because of potential negative genetic correlations among traits, there were still large gains (Figure 2). The traits that make up the selection index showed a wide range of heritability (0.05–0.74; Table S2), based on initial heritability estimates made from parent offspring regression in the F_1_ and based on multigenerational estimates (Table S2). Selection intensity between generations varied, ranging from 5–34% for different populations. The response to selection was close to the expectation based on the breeder’s equation [48]. With respect to yield traits, the first generation of selection for largest head diameter exceeded the breeder’s equation, while the second generation of selection slightly underperformed expectations. Also, individual seed weight underperformed in the first generation, but then exceeded expectation in the second generation.

One year of selection was sufficient to make progress toward the standard domestication ideotype. In addition to the direct selection on branching, head size and yield, indirect selection for decreased dormancy was incorporated by directly seeding plants into plots. Note that this slightly confounds our results, as we cannot be sure if gains were due to direct or indirect selection. Gains were apparent in the traits where selection was imposed (Figure 2; Figure S1). There were significant differences between each generation, e.g., non-overlapping confidence intervals of each generation, particularly for those traits that were part of the selection index, for example flower number (F_1_: 108 ± 5, IM_1_F_1_: 38 ± 8, IM_2_F_1_: 53 ± 15) and individual seed weight (F_1_: 0.05 ± 0.01, IM_1_F_1_: 0.07 ± 0.03, IM_2_F_1_:0.13 ± 0.02). The best lines (half-sib families) showed an increase in phenotype. For example, for largest head diameter (h^2^ = 0.58) the best IM_2_F_1_ line showed a 320% increase over the best F_1_ line. These phenotypic gains do not approach the domestic cultivated common sunflower, as the best intermated lines are still ~25–30% of our *H. annuus* elite control lines. However, with only two cycles of selection the observed gain is large when considering the head diameter of the wild perennial material is only ~5–10% that of the *H. annuus* cultivars. We did not observe a difference in selection efficiency for domestication syndrome traits (increased seed size) as compared to non-domestication traits (spreading ability). In both cases, selection was quite effective.

### 3.2. Perenniality Mapping in the Interspecific Population

*Helianthus tuberosus* is an autoallohexaploid likely formed from hybridization between *Helianthus hirsutus* (an autotetraploid of *Helianthus divaricatus*) and the diploid *Helianthus grosseserratus* [49]. The interspecific tetraploid domestication population used in this study was derived from crosses between the diploid *H. annuus* (three commercial inbred lines) and the autoallohexaploid *H. tuberosus* (wild collected from Rosemount, MN, USA). There has been a number of meiotic studies on *H. tuberosus* and of the interspecifc hybrids [50,51,52], meiosis within *H. tuberosus* is mixed polysomic and disomic, with a diploid portion of the genome and an autotetrploid portion of the genome. While there is limited evidence of crossing success between *H. annuus* and *H. grosseseratus* [53,54], it is still more likely of pairing between the same genome rather than different genomes. This means that three different subgenomes are present within each individual tetraploid hybrid (three sets of chromosomes from the two subgenomes of *H. tuberosus*, and one set of chromosomes from *H. annuus*). This has substantial implications for meiosis and the stability of fertility in subsequent generations and as the plant ages, as irregular pairing may lead to reduced fertility in both male and female, however this should stabilize over more generations of intermating.

The mapping analysis included 75 IM_1_F_1_ hybrids (*H. tuberosus* × *H. annuus*) segregating for tuber production and 12 founders of the population (11 wild collected *H. tuberosus* and one *H. annuus*-HA89 (merged from the technical replicates)). With respect to sequence data, there was uneven coverage across and within individuals (Figure S2). Variant calling was performed using two reference genomes of different *H. annuus* lines to maximize the potential for identification of useful markers. After filtering out loci with >30% missing data, <10% minor allele frequency, indels, and loci with segregation distortion, there were 224 markers called against the XRQ reference plus an additional 22 non-overlapping markers from the HA412-HO reference. These 246 markers were then subjected to a final LD filter (see Methods) and reordered, leaving 217 for use in QTL mapping.

**Figure 3:**
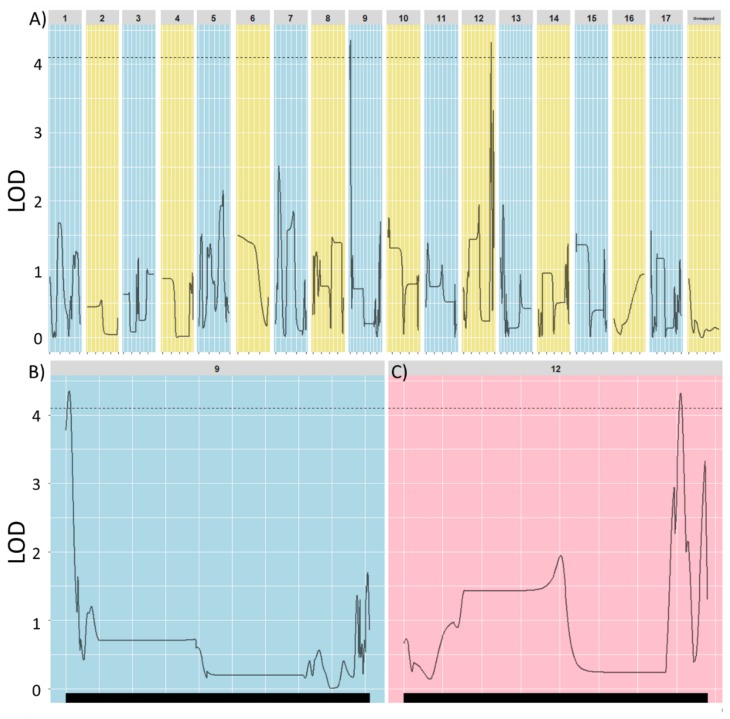
Quantitative trait locus (QTL) for tuber formation identified after markers placed on the HA412-HO genome, (**A**) Haley-Knott regression identified two significant QTL, implying oligogenic control across the seventeen chromosomes of *Helianthus annuus*; (**B**) Chromosome 9 close up and corresponding QTL; (**C**) Chromosome 12 close up and corresponding QTL. In all panels, the dotted line indicates a significance level of *p* < 0.01 as determined by a permutation test. LOD (logarithm of the odds).

Two significant QTLs were identified on linkage groups 9 and 12 that explained 21.9% and 21.5% of the phenotypic variance, respectively, for tuber presence-absence. (Figure 3). However, given the small size of the QTL mapping population, it is likely that estimates of QTL magnitudes are inflated and some QTLs were missed [55].

## 4. Discussion

### 4.1. Modified Domestication

Here we report that selection in interspecific populations produced large phenotypic gains in traits that are useful to agricultural systems. However, we cannot exclude drift and/or the potential for linked effects from the indirect selection of the direct seeding. There has been substantial interest in perennial sunflower as trap crop [56,57]. However, the process of domestication is long, even when the ideotype is known, and while we show progress toward towards a domestic ideotype, we recognize the observed populations are in the process of being domesticated and have not reached the desired ideotype. Interspecific crosses can have several potential outcomes, including: (1) outbreeding depression, in which hybrids are less viable or fertile than their parents [58]; (2) hybrid vigor or heterosis [59], in which hybrids have superior growth rates, biomass production, or yield compared to their parents; (3) transgressive segregation, in which hybrids exhibit extreme trait values (positive or negative) compared to either parent [60]; and (4) within plant segregation as a plant ages [61]. Hybrid vigor is typically strongest in first generation hybrids, where heterozygosity is maximized, whereas transgressive is most prominent in segregating hybrids. Outbreeding depression may be observed in first generation hybrids, later generation hybrids, or both.

It is not uncommon for multiple potential outcomes of hybridization to be visible in a single hybrid population [58]. For example, the interspecific F_1_ population described in this study exhibited heterosis for vegetative characters, as well as reduced fertility [31]. To build on this initial vigor, a strategy of intermating individuals to increase observed gain was used. This approach was used for two reasons: (1) to increase the amount of observable recombination [48], which may help identify transgressive sergeants; and (2) to increase fertility by increasing the frequency of meiotically compatible progeny. However, there is much progress still to be made in terms of generalizing the breeding of new crops and the utilization of new resources [62]. Positive domestication characteristics are often correlated, and we observed improvement in multiple characteristics simultaneously.

Within our interspecific breeding populations, the largest gains were observed for decreased branching and increased head size. This is not surprising, given that these traits had higher estimated heritability than yield or individual seed weight. Deviation from the breeder’s equation implies that the initial heritability estimate was not correct or that heritability changed between generations; both of these are possible due to the complex interspecific background of the population, which has yet to stabilize. While we did not yet observe dramatic increases in seed set, there were slight increases in the number of seeds per head, which may be a byproduct of increases in head size. The current selection index weights certain traits (e.g., head size) more than branching, since the perennial domesticated ideotype remains unclear. Thus, variation for both ideotypes can be maintained while still making phenotypic gains. In addition, it is possible to simultaneously select for multiple ideotypes - bifurcating the program to test the benefit of different plant architectures and phenology for ecosystem and food uses. Depending on whether phenotypic tradeoffs are present or not, it may also be possible to select for a combined ideotype in a single population. The wide phenotypic ranges provided ample opportunity for selection, as many families showed improved characteristics. With respect to the breeders’ equation, the results probably indicate that our estimates of selection intensity or heritability were slightly incorrect as opposed to a biological interpretation that assumes a different response for different traits. This demonstrates how domestication may have provided opportunities for both direct and indirect selection based on harvest/planting technology by early farmers, potentially leading to rapid phenotypic change in short time frames. To this end, for future generations of selection, we have decided to limit the number of characters, focusing on fertility-related traits.

### 4.2. Genetics of Tuber Formation

Understanding the genetic basis of tuberization in sunflower would be of great utility to a perennial sunflower breeding program. Initially, given the high heritability of tuber formation and the apparent segregation patterns of 1:3 in our IM_1_F_1_ population (a ratio typically associated with segregation of a single recessive locus), we thought it might be possible to map this trait in our population (the other domestication traits of interest exhibited phenotypic distributions that were too quantitative to map with this small population). However, our results instead suggest an oligogenic basis for tuber production (presence/absence) in our population. While the two significant QTLs jointly explain 43.4% percent variation explained (PVE), this estimate is likely inflated due to the Beavis effect. Also, we lacked power to detect additional modifier loci (if they exist) or to precisely define QTL intervals. Even given all these caveats, we were able to obtain flanking markers for the major ‘perenniality’ QTL, which can be used to facilitate further breeding effects. These results were similar to what has been observed in other species for perennial organ formation [63,64]. In the future, more precise mapping would be aided by a much larger mapping population and a modified genotyping by sequencing strategy such as that reported by Moyers et al. [65], in which two restriction enzymes and a duplex nuclease treatment were employed to reduce the proportion of high-copy fragments in GBS libraries.

## 5. Conclusions

Domestication can be viewed as accelerated evolution directed for human benefit. Well-defined objectives along with indirect objectives associated with the technologies used to grow/harvest/process may lead to rapid phenotypic change in short time frames and it is unclear when such progress will plateau. The most productive ideotype for a perennial domesticate is still unclear. However, using an ideotype similar to current domesticated annual crops, we have seen substantial progress in only a few cycles of selection for domestication syndrome traits. Despite the small mapping population, it was still possible to obtain marker-trait associations for tuber production. These markers will be assessed in further populations, to gain a better understanding of perennial and domestication syndrome traits. Here we see that neo-domestication may proceed rapidly, on much shorter timescales than the protracted domestication hypothesized for many species [66], in this case likely due to domesticated alleles being present in the population that could respond to targeted selection for domestication syndrome traits.

## Supplementary Materials

The following are available online at http://www.mdpi.com/2073-4425/9/9/422/s1, Figure S1: BLUP plotted vs line means with confidence intervals and prediction intervals (A) Average Head Diameter (B) Largest Head Diameter, and (C) Seed Per Head. The interior set of blue lines represents a 95% prediction interval and the outer set of red lines represents a 95% confidence interval. The breeding populations that are included are the initial wild parents, F_1_ plants, IM_1_F_1_ plants, and IM_2_F_1_ plants. Figure S2. (A) Sequencing depth across individuals, with red line indicating the median sequence coverage. Red line is the median number of sequences per line. (B) Reads within interspecific Hybrid 101B, a small number of sequences take up a disproportionate number of the reads. Table S1: Families and individuals used in this experiment. Table S2. Heritability estimates from BLUP and Kantar et al. [31].

## Data Archiving Statement

Data for this study are available at the NCBI sequence read archive: BioProject ID SRP127977.
