# Supplementary material for: Neo-Domestication of an Interspecific Tetraploid Helianthus annuus × Helianthus tuberous Population That Segregates for Perennial Habit
Source: Genes (Basel). 2018 Aug 21;9(9):422. doi: 10.3390/genes9090422 (PMC6162802; doi:10.3390/genes9090422)

Table S1. Families and individuals used in this experiment.

| | | | | | F1 parentage | |
|---|---|---|---|---|---|---|
| new designation based on generation | Designation | Generation | Designation-name | Previous Generation maternal parentage | maternal parent | paternal parent |
| MNPS_IM_SEL_7 | 370 | IM3F1 | JA215D 7-5 | JA215D for all generations | JA15 | HA89 |
| MNPS_IM_SEL_20 | 412 | IM3F1 | JA206D 3-10 | JA206D for all generations | JA6 | HA89 |
| MNPS_IM_SEL_4 | 339 | IM3F1 | JA213A 6-5 | JA213A for all generations | JA13 | HA89 |
| MNPS_IM_SEL_27 | 440 | IM3F1 | JA312E 11-15 | JA312E for all generations | JA12 | HA434 |
| MNPS_IM_SEL_12 | 388 | IM3F1 | JA207E 4-6 | JA207E for all generations | JA7 | HA89 |
| MNPS_IM_SEL_16 | 401 | IM3F1 | CMS210B 2-3 | CMS210B for all generations | CMS HA 89 | JA10 |
| MNPS_IM_SEL_29 | 448 | IM3F1 | JA216B 7-8 | JA216B for all generations | JA16 | HA89 |
| MNPS_IM_SEL_22 | 419 | IM3F1 | JA204A 3-4 | JA204A for all generations | JA4 | HA89 |
| MNPS_IM_SEL_8 | 371 | IM3F1 | JA215D 7-5 | JA215D for all generations | JA15 | HA89 |
| MNPS_IM_SEL_2 | 330 | IM3F1 | JA 211 A 5-16 | JA211A for all generations | JA11 | HA89 |
| MNPS_IM_SEL_14 | 394 | IM3F1 | JA2-2B 3-3 | JA2 for all generations | JAJA2 | HA89 |
| MNPS_IM_SEL_9 | 383 | IM3F1 | JA207C 4-5 | JA207C for all generations | JA07 | HA89 |
| MNPS_ROS_SEL1 | JA213_A 6-5.3 | IM2F1 | JA213A | JA213A for all generations | JA13 | HA89 |
| MNPS_ROS_SEL2 | JA_207C 4.5 | IM2F1 | JA207C | JA207C for all generations | JA7 | HA89 |
| MNPS_ROS_SEL3 | JA213_E 6-9 | IM2F1 | JA213E | JA213E for all generations | JA13 | HA89 |
| MNPS_ROS_SEL4 | JA211B5 | IM2F1 | JA211B | JA211B for all generations | JA11 | HA89 |
| MNPS_ROS_SEL5 | 94e.1 | IM2F1 | JA 217 F | JA for all generations | JA17 | HA89 |
| MNPS_ROS_SEL6 | JA210A5 _11.2 | IM2F1 | JA210A | JA210A for all generations | JA10 | HA89 |
| MNPS_ROS_SEL7 | 96.a.1 | IM2F1 | JA 217 H | JA217 for all generations | JA17 | HA89 |
| MNPS_IM_SEL_1 | 325 | IM3F1 | JA308A 10-16 | JA308A for all generations | JA8 | HA434 |
| MNPS_ROS_SEL8 | 93 | IM3F1 | JA 217 E | JA217 for all generations | JA17 | HA89 |
| MNPS_ROS_SEL9 | JA211F5 _11.6 | IM2F1 | JA211F | JA211F for all generations | JA11 | HA89 |
| MNPS_ROS_SEL10 | JA212_E 5_11.2 | IM2F1 | JA212E | JA212E for all generations | JA12 | HA89 |
| MNPS_IM_SEL_21 | 415 | IM3F1 | JA205D 3-10 | JA205D for all generations | JA5 | HA89 |
| MNPS_IM_SEL_15 | 395 | IM3F1 | JA213D 6-8 | JA213D for all generations | JA13 | HA89 |
| MNPS_IM_POP2 | 7 | IM2F1 | CMS 212B, CMS 218B | population for all generations | HA89 | Population 1 |
| MNPS_IM_POP6 | 15 | IM2F1 | JA 208B, JA 208D | population for all generations | Population 2 | HA89 |
| MNPS_IM_POP1 | 5 | IM2F1 | CMS208A, CMS 208B, CMS 208C, CMS 208D, CMS 208E | population for all generations | HA89 | Population 3 |
| MNPS_IM_4 | 12 | IM2F1 | CMS208 A | CMS210B for all generations | CMS HA89 | JA10 |
| MNPS_IM_39 | 156 | IM2F1 | JA314 A | JA314A for all generations | JA14 | HA434 |

| | | | | | | |
|---|---|---|---|---|---|---|
| MNPS_IM_11 | 25 | IM2F1 | CMS208 B | CMS208B for all generations | HA89 | JA8 |
| MNPS_IM_1 | 3 | IM2F1 | CMS201 C | CMS201C for all generations | HA89 | JA1 |
| MNPS_IM_19 | 63 | IM2F1 | JA212 H | JA212H for all generations | JA12 | HA89 |
| MNPS_IM_21 | 75 | IM2F1 | JA214 G | JA214G for all generations | JA14 | HA89 |
| MNPS_IM_10 | 23 | IM2F1 | CMS218 B | CMS218B for all generations | HA89 | JA18 |
| MNPS_ROS_SEL11 | JA_314_ AC-21 | IM2F1 | JA314A | JA314A for all generations | JA14 | HA434 |
| MNPS_IM_1 | 3 | IM1F1 | CMS201C | CMS201C for all generations | HA89 | JA01 |
| MNPS_IM_2 | 7 | IM1F1 | CMS205B | CMS205B for all generations | HA89 | JA05 |
| MNPS_IM_3 | 8 | IM1F1 | CMS205C | CMS205C for all generations | HA89 | JA05 |
| MNPS_IM_4 | 12 | IM1F1 | CMS208A | CMS208A for all generations | HA89 | JA08 |
| MNPS_IM_5 | 13 | IM1F1 | CMS208B | CMS208B for all generations | HA89 | JA08 |
| MNPS_IM_6 | 14 | IM1F1 | CMS208C | CMS208C for all generations | HA89 | JA08 |
| MNPS_IM_7 | 15 | IM1F1 | CMS208D | CMS200D for all generations | HA89 | JA08 |
| MNPS_IM_8 | 17 | IM1F1 | CMS209A | CMS209A for all generations | HA89 | JA09 |
| MNPS_IM_9 | 21 | IM1F1 | CMS212B | CMS212B for all generations | HA89 | JA12 |
| MNPS_IM_10 | 23 | IM1F1 | CMS218B | CMS218B for all generations | HA89 | JA18 |
| MNPS_IM_11 | 25 | IM1F1 | JA201A | JA201A for all generations | JA01 | HA89 |
| MNPS_IM_12 | 29 | IM1F1 | JA201E | JA201E for all generations | JA01 | HA89 |
| MNPS_IM_13 | 31 | IM1F1 | JA201H | JA201H for all generations | JA01 | HA89 |
| MNPS_IM_14 | 35 | IM1F1 | JA205D | JA205D for all generations | JA05 | HA89 |
| MNPS_IM_15 | 38 | IM1F1 | JA206C | JA206C for all generations | JA06 | HA89 |
| MNPS_IM_16 | 40 | IM1F1 | JA206E | JA206E for all generations | JA06 | HA89 |
| MNPS_IM_17 | 41 | IM1F1 | JA206G | JA206G for all generations | JA06 | HA89 |
| MNPS_IM_18 | 54 | IM1F1 | JA211B | JA211B for all generations | JA11 | HA89 |
| MNPS_IM_19 | 63 | IM1F1 | JA212H | JA212H for all generations | JA12 | HA89 |
| MNPS_IM_20 | 74 | IM1F1 | JA214E | JA214E for all generations | JA14 | HA89 |
| MNPS_IM_21 | 75 | IM1F1 | JA214G | JA214G for all generations | JA14 | HA89 |
| MNPS_IM_22 | 77 | IM1F1 | JA215B | JA215B for all generations | JA15 | HA89 |
| MNPS_IM_23 | 79 | IM1F1 | JA215D | JA215D for all generations | JA15 | HA89 |
| MNPS_IM_24 | 90 | IM1F1 | JA217B | JA217B for all generations | JA17 | HA89 |
| MNPS_IM_25 | 91 | IM1F1 | JA217C | JA217C for all generations | JA17 | HA89 |
| MNPS_IM_26 | 93 | IM1F1 | JA217E | JA217E for all generations | JA17 | HA89 |

| | | | | | | |
|---|---|---|---|---|---|---|
| MNPS_IM_27 | 94 | IM1F1 | JA217F | JA217F for all generations | JA17 | HA89 |
| MNPS_IM_28 | 96 | IM1F1 | JA217H | JA217H for all generations | JA17 | HA89 |
| MNPS_IM_29 | 101 | IM1F1 | JA218E | JA218E for all generations | JA18 | HA89 |
| MNPS_IM_30 | 114 | IM1F1 | JA305A | JA305A for all generations | JA05 | HA434 |
| MNPS_IM_31 | 121 | IM1F1 | JA306E | JA306E for all generations | JA06 | HA434 |
| MNPS_IM_32 | 129 | IM1F1 | JA307F | JA307F for all generations | JA07 | HA434 |
| MNPS_IM_33 | 139 | IM1F1 | JA309C | JA309C for all generations | JA09 | HA434 |
| MNPS_IM_34 | 140 | IM1F1 | JA309D | JA309D for all generations | JA09 | HA434 |
| MNPS_IM_35 | 142 | IM1F1 | JA309F | JA309F for all generations | JA09 | HA434 |
| MNPS_IM_36 | 143 | IM1F1 | JA309G | JA309G for all generations | JA09 | HA434 |
| MNPS_IM_37 | 148 | IM1F1 | JA312B | JA312B for all generations | JA12 | HA434 |
| MNPS_IM_38 | 151 | IM1F1 | JA312E | JA312E for all generations | JA12 | HA434 |
| MNPS_IM_39 | 156 | IM1F1 | JA314A | JA314A for all generations | JA14 | HA434 |
| MNPS_IM_40 | 164 | IM1F1 | JA315C | JA315C for all generations | JA15 | HA434 |
| MNPS_IM_41 | 173 | IM1F1 | JA317A | JA317A for all generations | JA17 | HA434 |
| MNPS_IM_42 | 185 | IM1F1 | JA318F | JA318F for all generations | JA18 | HA434 |
| MNPS_IM_POP1 | 5 | IM1F1 -bulk | CMS 208A, CMS 208B, CMS 208C, CMS 208D, CMS 208E | population for all generations | HA89 | Populati on 1 |
| MNPS_IM_POP2 | 7 | IM1F1 -bulk | CMS 212B, CMS 218B | population for all generations | HA89 | Populati on 2 |
| MNPS_IM_POP3 | 9 | IM1F1 -bulk | JA 201A, JA 201B, JA 201C, JA 201D, JA 201E, JA 201F, JA 201H | population for all generations | Populatio n 3 | HA89 |
| MNPS_IM_POP4 | 10 | IM1F1 -bulk | JA 202A, JA 202B | population for all generations | Populatio n 4 | HA89 |
| MNPS_IM_POP5 | 13 | IM1F1 -bulk | JA 206 A, JA 206B, JA 206C, JA 206D, JA 206E, JA 206G, JA 206H | population for all generations | Populatio n 5 | HA89 |
| MNPS_IM_POP6 | 15 | IM1F1 -bulk | JA 208B, JA 208D | population for all generations | Populatio n 6 | HA89 |
| MNPS_IM_POP7 | 23 | IM1F1 -bulk | JA 216A, JA 216B, JA 216C, JA 216D, JA 216E, JA 216F, JA 216G | population for all generations | Populatio n 7 | HA89 |
| MNPS_IM_POP8 | 37 | IM1F1 -bulk | JA 313 A, JA 313 B | population for all generations | Populatio n 8 | HA434 |
| MNPS_IM_SEL_1 | 325 | IM2F1 | JA308A 10-16 | JA308A for all generations | JA08 | HA434 |
| MNPS_IM_SEL_2 | 330 | IM2F1 | JA 211 A 5-16 | JA211A for all generations | JA11 | HA89 |
| MNPS_IM_SEL_3 | 338 | IM2F1 | JA213A 6-5 | JA213A for all generations | JA13 | HA89 |
| MNPS_IM_SEL_4 | 339 | IM2F1 | JA213A 6-5 | JA213A for all generations | JA13 | HA89 |
| MNPS_IM_SEL_5 | 368 | IM2F1 | JA213A 6-5 | JA213A for all generations | JA13 | HA89 |
| MNPS_IM_SEL_6 | 369 | IM2F1 | JA213B 6-9 | JA213A for all generations | JA13 | HA89 |
| MNPS_IM_SEL_7 | 370 | IM2F1 | JA215D 7-5 | JA215A for all generations | JA15 | HA89 |
| MNPS_IM_SEL_8 | 371 | IM2F1 | JA215D 7-5 | JA215A for all generations | JA15 | HA89 |

| | | | | | | |
|---|---|---|---|---|---|---|
| MNPS_IM_SEL_9 | 383 | IM2F1 | JA207C 4-5 | JA207A for all generations | JA07 | HA89 |
| MNPS_IM_SEL_10 | 385 | IM2F1 | JA308A 10-14 | JA308A for all generations | JA08 | HA434 |
| MNPS_IM_SEL_11 | 387 | IM2F1 | JA211A 5-13 | JA211A for all generations | JA11 | HA89 |
| MNPS_IM_SEL_12 | 388 | IM2F1 | JA207E 4-6 | JA207A for all generations | JA11 | HA89 |
| MNPS_IM_SEL_13 | 390 | IM2F1 | JA205C 3-11 | JA205A for all generations | JA07 | HA89 |
| MNPS_IM_SEL_14 | 394 | IM2F1 | JA2-2B 3-3 | JA2A for all generations | JA-5 | HA89 |
| MNPS_IM_SEL_15 | 395 | IM2F1 | JA213D 6-8 | JA213A for all generations | JA-2 | HA89 |
| MNPS_IM_SEL_16 | 401 | IM2F1 | CMS210B 2-3 | JA210A for all generations | HA89 | JA13 |
| MNPS_IM_SEL_17 | 408 | IM2F1 | JA201F 2-17 | JA201A for all generations | JA01 | HA89 |
| MNPS_IM_SEL_18 | 409 | IM2F1 | JA201F 2-17 | JA201A for all generations | JA01 | HA89 |
| MNPS_IM_SEL_19 | 411 | IM2F1 | JA206D 3-10 | JA206A for all generations | JA06 | HA89 |
| MNPS_IM_SEL_20 | 412 | IM2F1 | JA206D 3-10 | JA206A for all generations | JA06 | HA89 |
| MNPS_IM_SEL_21 | 415 | IM2F1 | JA205D 3-10 | JA205A for all generations | JA05 | HA89 |
| MNPS_IM_SEL_22 | 419 | IM2F1 | JA204A 3-4 | JA204A for all generations | JA04 | HA89 |
| MNPS_IM_SEL_23 | 421 | IM2F1 | JA316B 12-11 | JA316A for all generations | JA16 | HA434 |
| MNPS_IM_SEL_24 | 429 | IM2F1 | 318F 15-11 | JA318A for all generations | JA18 | HA434 |
| MNPS_IM_SEL_25 | 430 | IM2F1 | JA316B 12-11 | JA316A for all generations | JA16 | HA434 |
| MNPS_IM_SEL_26 | 433 | IM2F1 | JA308A 10-7 | JA308A for all generations | JA08 | HA434 |
| MNPS_IM_SEL_27 | 440 | IM2F1 | JA312E 11-15 | JA312A for all generations | JA12 | HA434 |
| MNPS_IM_SEL_28 | 447 | IM2F1 | JA206E 1-7 | JA206A for all generations | JA06 | HA89 |
| MNPS_IM_SEL_29 | 448 | IM2F1 | JA216B 7-8 | JA216A for all generations | JA16 | HA89 |

*Population 1 is composed of full sib individuals with that are descended from JA8 and CMS HA89

*Population 2 is composed of half sib individuals with that are descended from JA12, JA18, and CMS HA89

*Population 3 is composed of full sib individuals with that are descended from JA1 and HA89

*Population 4 is composed of full sib individuals with that are descended from JA2 and HA89

*Population 5 is composed of full sib individuals with that are descended from JA6 and HA89

*Population 6 is composed of full sib individuals with that are descended from JA8 and HA89

*Population 7 is composed of full sib individuals with that are descended from JA16 and HA89

*Population 3 is composed of full sib individuals with that are descended from JA13 and HA434

Table S2. Heritability estimates from BLUP and Kantar et al., 2014

| Trait | BLUP heritability | Kantar et al., 2014 |
| --- | --- | --- |
| Average Head Diameter | 0.74 | 0.67 |
| Largest Head Diameter | 0.58 | 0.48 |
| Seeds Per Head | 0.23 | 0.31 |
| Seed Weight | 0.05 | 0.16 |
| Individual seed weight | 0.43 | 0.22 |

*Figure S1. BLUP plotted vs line means with confidence intervals and prediction intervals A) Average Head Diameter B) Largest Head Diameter, and C) Seed Per Head. The interior set of blue lines represents a 95% prediction interval and the outer set of red lines represents a 95% confidence interval. The breeding populations that are included are the initial wild parents, $F_1$ plants, $IM_1F_1$ plants, and $IM_2F_1$ plants.*

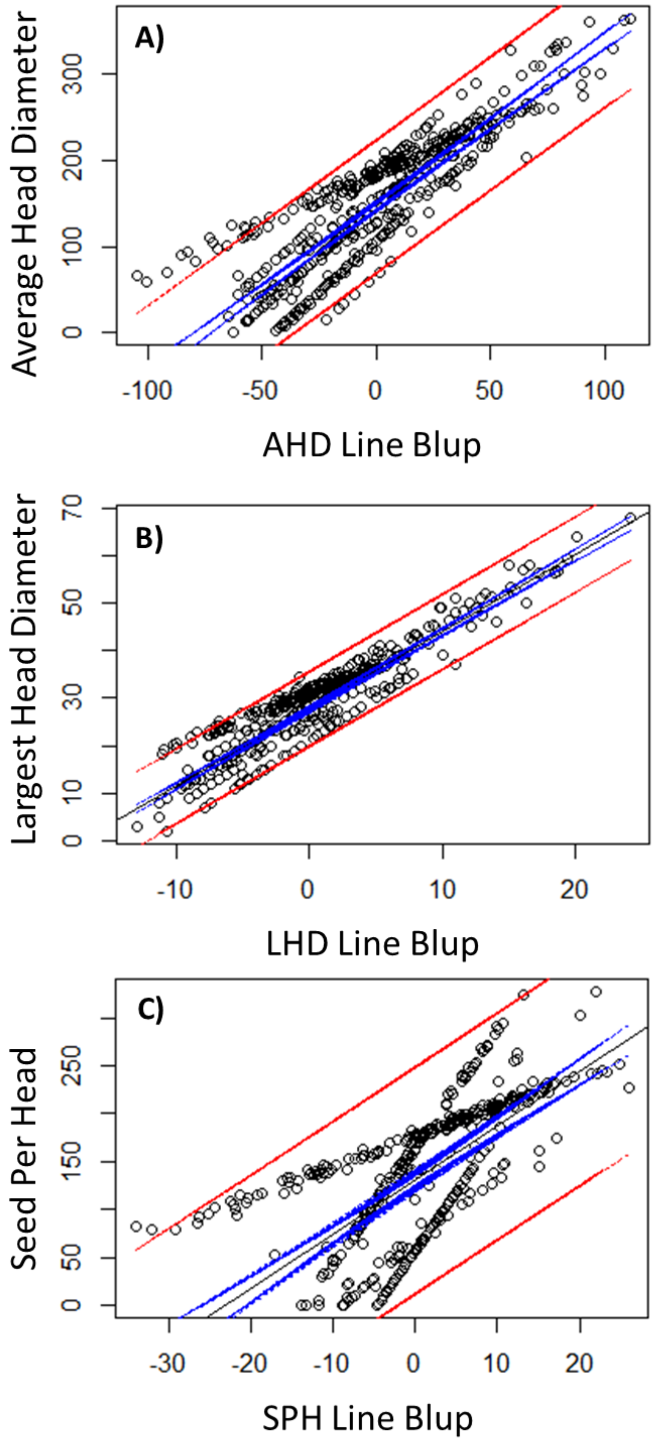

*Figure S2. A) Sequencing depth across individuals, with red line indicating the median sequence coverage. Red line is the median number of sequences per line. B) Reads within interspecific Hybrid 101B, a small number of sequences take up a disproportionate number of the reads.*

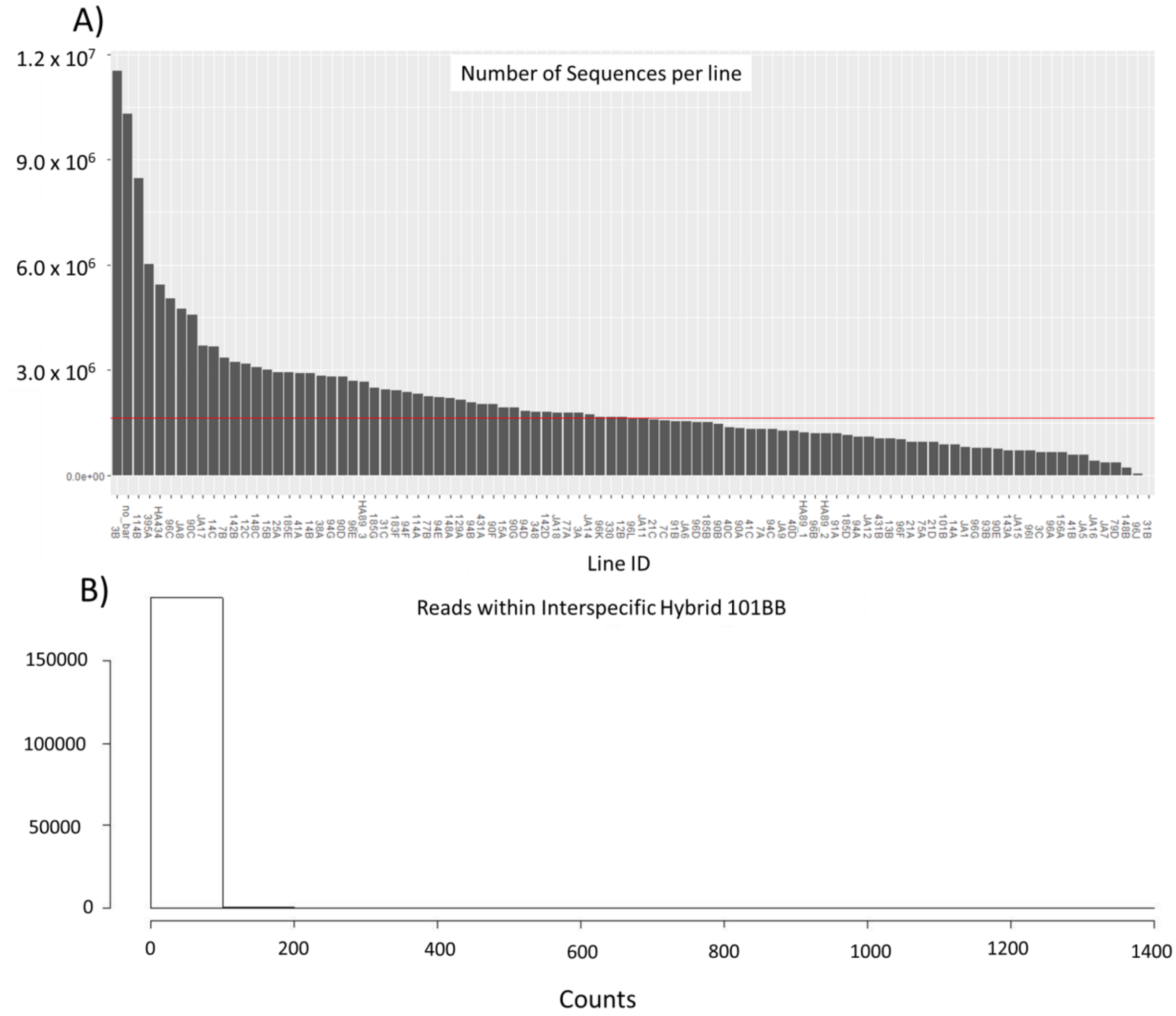

Supplement: Supplementary file 1 [file genes-09-00422-s001.pdf]
